# Time-Driven, Activity-Based Costing to Reduce Interventional Radiology Suite Idle Time

**DOI:** 10.7759/cureus.31862

**Published:** 2022-11-24

**Authors:** Mohammad Ghasemi Rad, David Wynne, Mahan Ghasemi, Christie Lincoln, Cliff Whigham

**Affiliations:** 1 Department of Radiology, Baylor College of Medicine, Houston, USA; 2 Undergraduate Student, Texas A&M School of Veterinary Medicine & Biomedical Sciences, College Station, USA

**Keywords:** healthcare, time-driven activity-based costing, idle time, cost reduction, interventional radiology

## Abstract

Background

With the ever-increasing complexity of today’s healthcare environment, it is evident that there is a higher demand to deliver high-quality, accessible, efficient, and affordable healthcare. At the same time, these changes are accompanied by decreasing rates of reimbursement. This can be attributed to the shift from fee-for-service to value-based payment methods in the industry. The reception of such changes in the appropriate manner is crucial to improvement and the much-demanded reform in our healthcare system. To adapt to this changing landscape, hospitals and healthcare systems must incorporate proper measures to identify extraneous spending, control costs, and streamline patient care. Our goal in this study was to use the time-driven, activity-based costing (TDABC) model to quantify the costs at every step as an inpatient goes through the care process in an interventional radiology department.

Methodology

After identification and mapping of all the steps involved from interventional radiology (IR) consult placement to patient transport to the postoperative recovery area, time data were collected for each step of the process. One of the steps was then selected for intervention. Our focus was on the time interval between one patient leaving after a completed procedure and the next scheduled patient entering the IR suite (heretofore referred to as idle time). To decrease the idle room time between patients, the interventional radiologists, IR administrations, nurse manager, transportation manager, and charge nurse first met as a group to set a realistic initial goal. Pre-intervention data were collected.

Results

After the collection of pre-intervention data, the average idle time of the IR suite was found to be 40 minutes. After a multidisciplinary discussion, our goal was to reduce this time to 25 minutes. Post-intervention data found the average time decreased to 24 minutes. Calculation of average costs per unit time for staff, IR room, and equipment yielded an approximate cost of $57 per minute of time in the IR suite.

Conclusions

Considering the near 40% decrease in suite idle time as well as the cost per minute of material, equipment, and staff (at ~80% capacity), this study proves that the TDABC system is a viable method of targeting bottlenecks in operations and streamlining patient care by reducing costs while optimizing the process patients go through during care continuum.

## Introduction

With the ever-increasing complexity of today’s healthcare climate, it is evident that there is a higher demand for accountability toward delivering high-quality, accessible, and affordable healthcare. At the same time, we see these changes accompanied by decreasing rates of reimbursement. This can be attributed to the shift from fee-for-service to value-based payment methods in the industry. The reception of such changes in the appropriate manner is crucial to improvement and the much-demanded reform in our healthcare system. To ensure a successful transition, we need to incorporate proper measures to identify extraneous spending, control costs, and streamline patient care [1].

Within healthcare in the United States, practitioners and administrations jointly seek to deliver the highest quality of care possible at the lowest cost manageable. This goal is attainable through coordinated efforts and systemic changes that can be implemented using a calculated approach such as time-driven, activity-based costing (TDABC). TDABC is a method, put forth by Robert Kaplan and Steven Anderson, by which the step-by-step process through which a patient goes in their course of healthcare can be mapped out and a cost per unit time of each employee, equipment, and material can be calculated [2]. Subsequently, through proper intervention, this process can be fine-tuned by identifying bottlenecks, inefficiencies, and non-necessities and eliminating them to streamline care while simultaneously cutting costs. Proper reallocation of resources based on the time each step of the process takes while considering the cost per unit time of the operation can be crucial to hospital systems given the shift in healthcare reimbursement from a fee-for-service to a value-based care system [3].

The goal of this study was to carry out a TDABC plan in making changes to the interventional radiology (IR) suite at Ben Taub Hospital, part of the Harris Health System, to streamline healthcare for patients going through the interventional radiology department, as well as to reduce the healthcare/hospital cost and improve patient satisfaction and room turnover.

## Materials and methods

**Figure 1:**
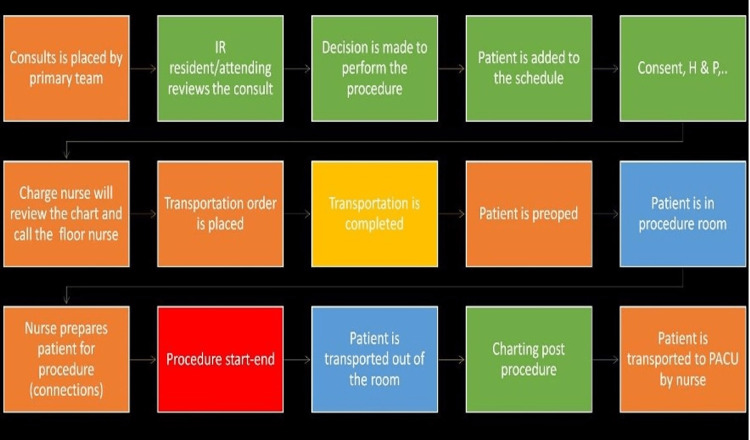
Steps taken in this study to implement time-driven, activity-based costing principles. IR: interventional radiology; PACU: post-anesthesia care unit

In the Ben Taub hospital, one of the two Harris Health System hospitals, there are two angiography suites (one monoplane and one biplane), two ultrasound rooms, of which one is dedicated to paracentesis, and one CT room used both for procedures and diagnostic CT examinations. Our study focused on the angiography rooms, especially the monoplane room; however, later in our study, both the biplane room and the main ultrasound room were included in the data collection. The pre-intervention period spanned from October 2020 to December 2020 which included a total of 61 patients. There are three dedicated body IR faculty, two neuro-interventional faculty, a physician assistant, and a nurse practitioner with four radiology residents at any time point rotating in the IR department. Each junior resident has a continuous three-month rotation, and a senior early-specialization in interventional radiology (ESIR) fourth-year radiology resident has a continuous two to four-month rotation. The resident on their first month of rotation is responsible for inpatient consents and notes as well as ultrasound procedures. The second-month resident is responsible for the CT-guided procedure and helping the first-month resident as needed. The third-month resident is responsible for neuro-interventional procedures and any interventional procedures done in the neuro room. Finally, the last resident is a senior (PGY-5) ESIR resident who supervises all residents and performs all body procedures. The initial step for us was to map out the individual stages of the process that the patient goes through before and after they are in the IR suite (Figure 1).

This includes initial consult placement by the referring clinical service, discussion with the IR resident and/or attending, and review of the patient’s electronic medical record and imaging. Subsequent steps include orders for any additional necessary pre-procedure laboratory testing, placing the patient on the schedule based on priority, nurse review of the patient’s chart, patient transportation to the preoperative holding area, the performance of the procedure, and postoperative stages and transitions. For effective management via TDABC, each of the above steps that the patient goes through would have to be associated with how much time and cost per unit time it takes for the process to work. As this study was a proof of concept, we focused on reducing the time it takes between a patient leaving the IR suite and the next patient entering; this, in turn, would reduce overall costs, as detailed below. The time data for each of the steps of this process were gathered and documented in a preprinted table by a designated IR technologist. The personnel cost was calculated by the median salaries of staff involved in the process. While gathering data about the time allotted to each task, it is important to account for the fact that the practice operates at 80-85% of full capacity when we consider time for breaks, arrival, departure, and communication. For equipment, time must be allocated for maintenance, repair, and scheduling fluctuations. After we consider the capacity at which personnel and machinery operate, we can assign costs per unit of time and determine how much each step of the process is costing us as well as how resources are being allocated to each step. The product of cost rates and the time spent in each step of the process will give us the total costs of this patient care cycle. The outlines below show the proper steps for adapting a TDABC system to better streamline patient care in a practice, as well as the specific steps taken in this study to apply the concept and prove the functionality thereof. Given, this study was strictly quality improvement, institutional review board (IRB) approval was not required per our institutional policy.

Pre-intervention

**Figure 2:**
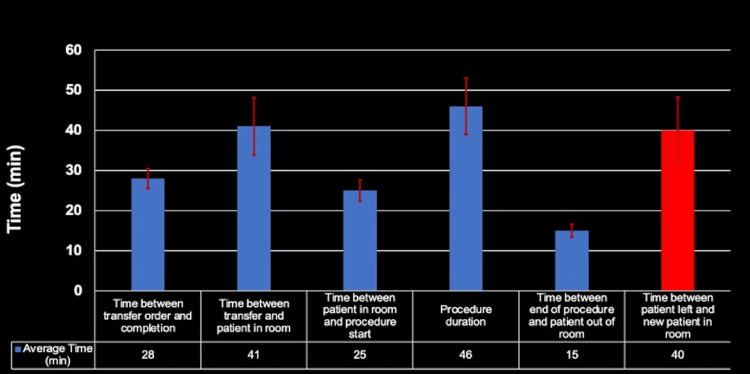
Pre-intervention bar chart demonstrating the time spent in each step.

After gathering data on each step, a chart was made and time data regarding each procedure were entered by a dedicated IR technologist. Our study focused on inpatient costs associated with the IR procedure from patient transportation to the IR department through post-procedure cleaning of the room and transporting the patient to a recovery area. The data were collected over a two-month period from October 15 to December 15. Data were analyzed to determine the bottleneck of the operation with mean, standard deviations, and confidence intervals (Figure 2).

Multiple meetings were conducted by IR staff that concluded that room idle time should be the initial focus given the extended period of average lag time between the patient leaving the room and the next patient entering the room. The average time it took for a patient to be transported at the end of procedures, room cleaning, and the transportation of the next patient entering the room was 40 minutes. The inpatient costs associated with this process were of importance to the study. Considering employee salaries, cost of instruments, and consumables at the capacity of normal operation, the cost per unit time was calculated to be $57/minute. The goal of this project, therefore, was to reduce the average time for room idle time from 40 minutes to 25 minutes, which would save Ben Taub Hospital an average of $855 for every room turnover.

## Results

Intervention and challenges

**Figure 3:**
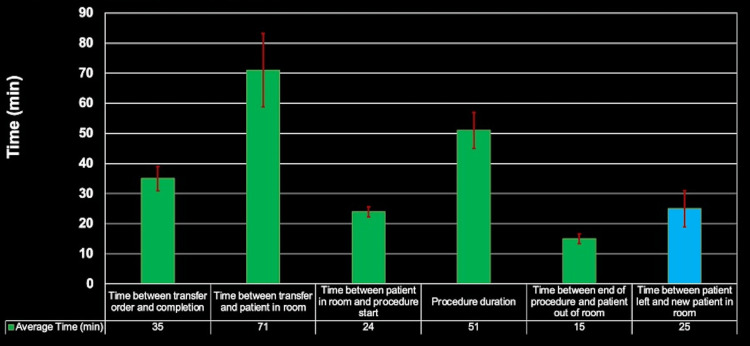
Post-intervention bar chart demonstrating the time spent in each step.

The changes that needed to be implemented to prove that TDABC is an effective approach for optimizing patient care and costs to practice are systemic and involve multiple stakeholders. The full potential of TDABC can only be realized with sufficient time and personnel resources needed to conduct a detailed outline and analysis of time, supplies, costs, and general workflow. Nonetheless, the IR physicians and administration as well as nursing leadership coordinated to accomplish the goals of this study. The intervention included hiring more nurses and allocating them to the preoperative area, opening a new IR post-anesthesia care unit with more capacity (six beds, previously two), as well as some procedural changes to the preoperative process. These changes included handoff occurring prior to the transport order (which was better accomplished with the hiring of more nurses) and completion of informed consent and physician preprocedure documentation preoperatively to streamline and accelerate the preoperative process Additionally, strict adherence to a 7:00 am start time for the first procedure of the day (7:15 am on days with scheduled support from the anesthesiology service), as well as improvement in communication between physician and nursing staff regarding the next patient and an alternative (one or two) aided efficiency. With this coordinated team effort, systemic alterations to the preoperative process were made, and the average time for IR suite turnover was again surveyed. A highlight of this study is that data from the IR suite were collected over three months (Figure 3) and then continued for two years.

Post-intervention

The collection of new data for two years yielded expected remarkable results with regard to time saved. The goal of the study was to reduce the time between the previous patient and the new patient in the room from 40 minutes to 25 minutes to reduce the costly idle time of the IR suite. This objective was accomplished as the post-intervention data showed an average time of 24 minutes for IR suite turnover, thus proving TDABC to be a functional method with which we can reduce costs to the practice, while simplifying, expediting, and overall improving the care provided to patients.

## Discussion

In an earlier study by Hauser et al., TDABC methodologies proved effective in reducing the costs of central venous catheter (CVC) placement in the IR department. A flow chart was used to map the steps from scheduling CVC placement all the way to patient discharge or transportation. Compartments of the chart were color-coded based on specific personnel involved. Costs were calculated based on the amount of time physicians, nurses, and technologists spent in the process and their respective capacity cost rates. Equipment, disposables, space, and other fixed costs were also accounted for. Due to the high-volume nature of this procedure, pre- and post-operational care times and costs were assumed to be standard rather than directly measured, but using the capacity cost rates of different personnel allowed for experimentation with different providers for the procedure. In this study, the use of procedural advanced practice providers instead of the deployment of IR attending physicians proved to be effective at cutting costs from an average of $904.65 per CVC placed to $856.30. Mapping out the steps of the procedure as well as variable and fixed costs using the TDABC model also meant identifying factors that cannot be changed and/or will make no difference to costs if varied [4]. A similar study was performed in the Texas Children Hospital to compare portable and in-suite tunneled femoral line placement in pediatrics that did not demonstrate any change in cost based on the TDABC algorithm [5]. Another study used TDABC to evaluate the use of magnetic resonance high-intensity focused ultrasound in the treatment of cancer-related bone pain [6].

In another study by Lewis et al. [7], TDABC implementation focused specifically on thoracic duct embolization procedures in the IR department. The steps between patients entering the procedure room and leaving it were mapped out, and each phase of the process was color-coded to reflect which personnel were primarily responsible for it. The labor times spent by each personnel during the procedure were accounted for using TDABC methods, and other costs for labor, capital equipment, and disposable materials were determined across a range using multivariate sensitivity analysis. In determining cost rates for personnel and material, practical capacity, defined as the amount of time a resource is available, as well as cost capacity rates were used in determining every resource’s cost to the practice. The variation in wage was found to have minimal effect on the variation in total cost. Univariate sensitivity analysis was used to determine that the major contributor to rising costs was disposable items. Specifically, the cyanoacrylate glue and selected embolic coils were the two most costly factors for the practice referring to thoracic duct embolization procedures. Using TDABC to identify where the most inevitable costs originated, the team was able to experiment with alternative tissue adhesives and limit the usage of costly ones to decrease costs [7]. Ljuboja et al. used TDABC to compare transarterial chemoembolization, transarterial radioembolization, and ablation for patients presenting with hepatocellular carcinoma. This demonstrated that the ablation cost was significantly less than the transarterial technique for hepatocellular carcinoma [8]. Another study by Martin et al. used TDABC for colonoscopy, carpal tunnel syndrome, and aortic valve replacement. The study concluded that TDABC was associated with decreased costs associated with each case [9].

In the American College of Radiology, there is yet another study on the importance and efficacy of TDABC implementation in healthcare settings to increase efficiency toward high-value patient care. This study by Shankar et al. highlights the importance of decreasing the time patients spend in the emergency department which, in turn, increases the turnover in the emergency room in response to the increased influx of patients. This study focused on the oral administration of contrast solution for patients presenting to the emergency department with nontraumatic abdominal pain [10]. By mapping out both the time-related and monetary costs of the procedures revolving around abdominal CT scans, the team was able to explore options regarding policy changes for the institution regarding the criteria needed to be met for an oral contrast solution to be administered as opposed to an intravenous contrast solution. The concern being addressed by the study was that oral contrast can lead to the delineation of the bowel loops and take up more time, though it is usually more effective in diagnosing gastrointestinal pathologies. A portion of the study was a retrospective assessment of electronic medical records of previously performed contrast-enhanced abdominopelvic CT scans. Rather than interviews with personnel, as was done in our study, this study mapped the oral contrast solution administration process through prospective observation of a complete care cycle. Direct estimates of time spent for each step were made and indirect estimates were made through staff interviews, whereas preprinted tables were utilized in our study to get a sense of the time each step takes. Labor costs were accounted for using TDABC methodologies, accounting for staff salaries, capacity costs, and time allocated by each staff to perform tasks in the above-referenced studies and our own. Material costs included non-negligible costs and materials, both disposable and non-disposable. The intervention in this study was the change in policy regarding who receives oral contrast solution. The policy changed so that oral contrast was only necessary if a patient’s body mass index is over 25 kg/m^2^, the patient has had recent surgery, or the patient has had a history of inflammatory bowel disease. The results of this study showed a 52% decrease in annual costs for the practice. The difference in cost originated from monetary cost differences between oral and intravenous contrast solution administration, yet time comparisons of barium sulfate, diatrizoate meglumine-sodium, and intravenous administration yielded no significant differences. However, this study also serves to prove that through multidepartmental cooperation, cost analysis, and proactive observation, TDABC methodologies can be used to cut down costs to practice and, in turn, increase the value of care [10]. Kohler et al. used TDABC to evaluate endoscopic versus open surgery for the treatment of carpal tunnel syndrome. Based on their results, endoscopic carpal tunnel release was 44% more expensive than open carpal tunnel release [11].

These studies exhibit how such methodologies can be used to identify and evaluate the main cost drivers of a practice. During the process of using TDABC in a healthcare setting, each study, including ours, proved it is essential to dedicate time and resources and take initiative to properly map out every step of a care cycle for patients. In some settings, where the procedure under focus is part of many broader processes of care, it can be more challenging to accurately map out the steps a patient takes before and after the operation, and, therefore, it may present more extraneous factors into time and costs allocated to the procedure based on patient diagnoses. However, the motif of accurate mapping of a care process being essential prior to intervention remains. Next, these studies testify to the importance of accounting for capacity when calculating costs. After the proper mapping and calculations regarding a care cycle are complete, intervention toward increasing value can occur. Value can be increased with the decrease in costs and time spent which, in turn, benefits the practice. While these studies all had varied approaches to cutting down on costs and increasing the value and quality of care, they showed that through TDABC methods and data collection, a practice can pinpoint the source of unnecessary costs and tackle bottlenecks in their process of patient care [1,12-15]. While our study served as a proof of concept, focusing on the idle time of the IR suite and the costs associated with it, we hope to expand TDABC to other departments and portions of patient care to further reduce bottlenecks and inevitable costs while increasing the value of care we provide to patients. TDABC is a means of understanding all the inputs into a process, such as time, money, and resources, to better streamline the output, which cannot be accomplished without the inclusion of many different factors in determining costs and times.

**Figure 4:**
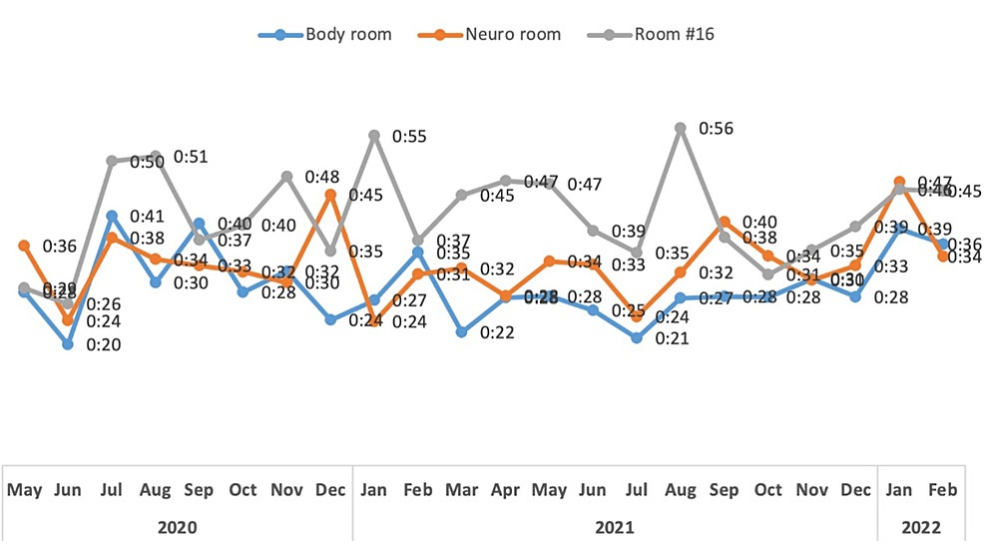
Average room turnover for three rooms (May 2020 to February 2022).

It is important to note that while multiple processes and stages of the care cycle were mapped and measured for time and resources allotted to them, this study only focused on one parameter as a way to refrain from overcomplicating the proof of concept, but with further efforts, more time, and more resources dedicated to data collection and proper measures, the principles of TDABC can be widely applied in multiple departments and tasks involved in patient care. We aim to identify and tackle all bottlenecks regarding the processes surrounding procedures in the IR suite, but as mentioned earlier, this study was conducted with a singular focus as opposed to a multiparameter TDABC study. This was to prove the efficacy of TDABC implementation, and by expanding on the positive outcomes we experienced in this study, we can further increase patient care value in the future through more time and resource dedication, as well as the involvement of more departments within the practice. During our study, the coronavirus disease 2019 (COVID-19) pandemic did not significantly affect our turnover time that has remained at 30 minutes for the body interventional room and 33 minutes in the neuro-interventional room (Figure 4).

This is because our team scheduled all COVID-19 patients, unless emergent, at the end of the day and back-to-back to avoid time-consuming terminal cleaning of the IR suite in between cases.

We aim to focus on the transportation time with a goal of decreasing it to 20 minutes, as well as the time between the patient entering the room and the start of the procedure, and the time between the end of the procedure end and the patient leaving the room. Though the COVID-19 pandemic did pose many challenges and extraneous circumstances, data collection and monitoring continued.

Limitations

One of the challenges in this study was the fact that Ben Taub Hospital is a non-profit, government entity, so changes are more difficult to execute because nurses and technologists are controlled directly by the administration. There are also frequent nurse and resident turnovers. The COVID-19 pandemic occurred in the middle of our study and added another layer to our study that was not anticipated.

## Conclusions

Considering the near 40% decrease in suite idle time as well as the cost per minute of material, equipment, and staff (at ~80% capacity), this study proves that the TDABC system is a viable method of targeting bottlenecks in operations and streamlining care for patients by reducing costs while optimizing the process patients go through during care continuum.
